# Unveiling the unexpected sinking and embedding dynamics of surface supported Mo/S clusters on 2D MoS_2_ with active machine learning

**DOI:** 10.1002/smo.20240018

**Published:** 2024-08-08

**Authors:** Luneng Zhao, Yanhan Ren, Xiaoran Shi, Hongsheng Liu, Zhigen Yu, Junfeng Gao, Jijun Zhao

**Affiliations:** ^1^ State Key Laboratory of Structural Analysis for Industrial Equipment&School of Physics Dalian University of Technology Dalian China; ^2^ Institute of High Performance Computing (IHPC) Agency for Science, Technology and Research(A*STAR) Singapore Singapore

**Keywords:** active learning, machine learning potential, Monte Carlo, surface‐supported clusters

## Abstract

Surface‐supported clusters forming by aggregation of excessive adatoms could be the main defects of 2D materials after chemical vapor deposition. They will significantly impact the electronic/magnetic properties. Moreover, surface supported atoms are also widely explored for high active and selecting catalysts. Severe deformation, even dipping into the surface, of these clusters can be expected because of the very active edge of clusters and strong interaction between supported clusters and surfaces. However, most models of these clusters are supposed to simply float on the top of the surface because ab initio simulations cannot afford the complex reconstructions. Here, we develop an accurate graph neural network machine learning potential (MLP) from ab initio data by active learning architecture through fine‐tuning pre‐trained models, and then employ the MLP into Monte Carlo to explore the structural evolutions of Mo and S clusters (1–8 atoms) on perfect and various defective MoS_2_ monolayers. Interestingly, Mo clusters can always sink and embed themselves into MoS_2_ layers. In contrast, S clusters float on perfect surfaces. On the defective surface, a few S atoms will fill the vacancy and rest S clusters float on the top. Such significant structural reconstructions should be carefully taken into account.

## Graphical abstract

By employing active machine learning potential into Monte Carlo simulations, we interestingly disclosed that Mo clusters always sink and embed themselves in MoS_2_ layers. In contrast, S clusters float on perfect surfaces. On the defective surface, a few S atoms fill the vacancy and rest S clusters float on the top. Such significant structural reconstructions should be carefully taken into account.

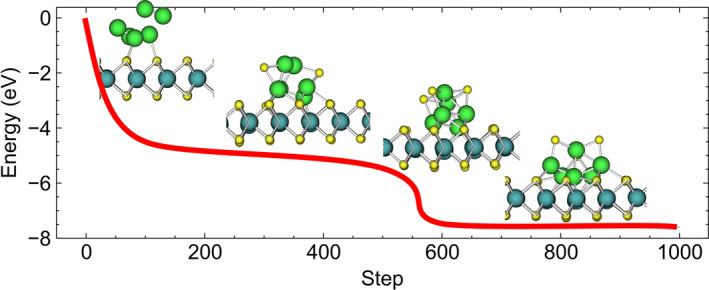

## 1 INTRODUCTION

Two‐dimensional materials have become a hot topic in research in recent years due to their unique properties and potential applications in areas such as electronics, optoelectronics, and energy storage.[^1^, ^2^, ^3^] Chemical vapor deposition (CVD) is a widely used experimental technique for the preparation of 2D materials, mainly by converting gaseous precursor substances into solid films or powders.[^4^, ^5^] However, some byproducts, such as excess elemental clusters, may deposit on the surface of the already grown 2D materials during the CVD process.^[6]^ Although theoretical and simulation studies have provided some insights, experimental results often deviate significantly from expectations.[^7^, ^8^] These discrepancies may arise from multiple factors, including the complexity and unpredictability of experimental conditions, as well as the incomplete alignment of some assumptions in theoretical models with actual circumstances. For example, various defects such as vacancy defects and adatom defects have been extensively reported,[^9^, ^10^, ^11^, ^12^, ^13^] but their influence on material properties, especially other possible configurations, remains incompletely understood and investigated. This limitation not only hinders our fundamental scientific understanding of 2D materials but also restricts their further development in practical applications.

Besides, single atom and single supported clusters have attracted lots of attention due to their high active and production selection for catalysts. Ab initio simulation is extensively used to explore the catalyst mechanism. In these studies, the single atom and single supported clusters are usually anchoring by the surface's defects. Or they are supposed to simply float on the top of the surface. However, the single atom and single clusters with nano size are very active, there may be very strong interaction between surface and supported atom/clusters, especially for transition metal clusters. Therefore, single atom and single supported clusters could etch the surface and anchor firmly. Severe deformation, even dipping into the surface, of these clusters can be expected. In recent years, the interaction between clusters and material surfaces has attracted considerable attention.[^14^, ^15^, ^16^, ^17^, ^18^] However, ab initio simulations cannot afford the complex reconstructions quickly, while classical molecular dynamics cannot deal with such complex bond recombination.

In recent years, machine learning has emerged as a powerful tool for investigating the properties of materials.[^19^, ^20^, ^21^, ^22^, ^23^, ^24^, ^25^] These models, trained on data from first principles calculations or experiments, are capable of efficiently and accurately determining various material properties [^26^, ^27^, ^28^, ^29^] A typical application of machine learning in materials simulation is the use of neural networks to fit interatomic potential energy surfaces, also known as machine learning potential (MLP).[^30^, ^31^, ^32^] These potentials can be employed in mechanical applications such as molecular structure optimization and molecular dynamics simulations.^[33]^ The utilization of MLPs enables efficient exploration of the material space's potential energy surface, thereby providing the potential to address the complex structural problems faced in surface clusters.

Although single‐atom decoration and perfect surfaces have been extensively studied,[^34^, ^35^, ^36^, ^37^] the behavior of clusters that naturally form during growth processes remains largely unexplored. These clusters can significantly influence the performance and properties of the material. MoS_2_ has attracted much attention for its exceptional catalytic and optoelectronic properties.[^38^, ^39^] However, there are still many mysteries regarding the interaction mechanisms between the surfaces and atomic clusters during growth processes. In particular, our understanding of the behavior of larger‐sized clusters on MoS_2_ surfaces, how they affect the growth process, and ultimately, how they influence the material's performance is limited. This knowledge is crucial for controlling the growth of MoS_2_ and optimizing its properties. To gain deeper insights into this matter, we combined machine‐learning potentials with Monte Carlo simulations (MLMC) to investigate the behavior of clusters on MoS_2_ surfaces. This approach allowed for a more comprehensive search of the configurational space, providing a more complete understanding of the interactions between clusters and surfaces in this important two‐dimensional material system.

Specifically, we focus on the interaction between Mo and S clusters (sizes 1–8) and the surface of single‐layer MoS_2_, as well as the point defects in MoS_2_. This involved examining the behavior of these clusters upon deposition on both perfect and defective MoS_2_ surfaces. In the case of perfect MoS_2_, S clusters exhibited minimal interaction with the surface upon deposition, showing only mild physical adsorption. However, due to Mo clusters' high surface energy, they readily incorporated themselves into the perfect MoS_2_ structure to lower the surface energy, thus forming a new class of defects. We also studied the behavior of Mo and S clusters on defective MoS_2_ surfaces. In the case of Mo‐V, a single S atom from an S cluster will readily occupy the vacancy site. For S clusters larger than a single atom, the additional S atoms will bond with the unsaturated S atoms surrounding the Mo‐V. For S_2_‐V, Mo clusters still embedded themselves in the surface as they would in the case of a perfect surface. Past investigations have often suggested that excess clusters deposited on the surface during CVD growth could be easily removed through post‐synthesis cleaning methods such as chemical etching or thermal annealing.[^34^, ^35^, ^36^, ^37^] However, our research indicates that the incorporation of Mo clusters into the MoS_2_ lattice at the atomic level may be irreversible, posing significant challenges to their removal. Therefore, it is crucial to exert stringent control over the Mo source during the CVD growth process to minimize the formation of these embedded Mo clusters. Additionally, optimizing the growth conditions to halt the supply of Mo source upon the completion of growth could further alleviate this issue. This will facilitate the production of high‐quality 2D materials with tailored properties suitable for advanced applications in electronics, optoelectronics, and catalysis.

## 2 METHOD

**FIGURE 1:**
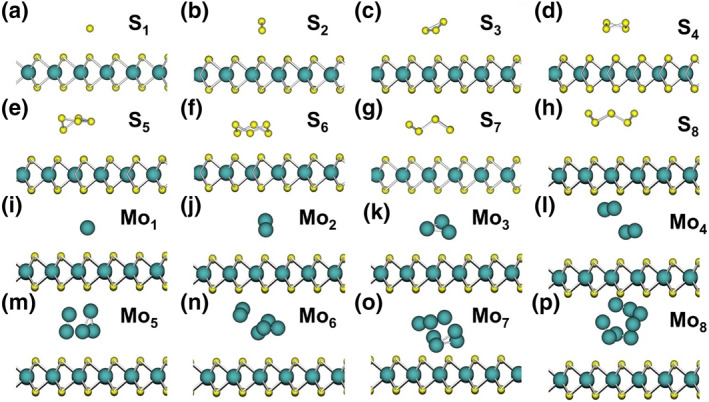
Initial data used for the first‐time fine‐tuning of the MLP model at the start of the active learning process.

In this study, the MLP model used is GemNet,^[40]^ a graph neural network capable of directly modeling crystal structures based on molecular graphs. We chose GemNet due to its exceptional ability to capture complex interactions and its successful applications in similar materials science contexts. Unlike traditional neural networks, its graph‐based approach allows for a more accurate representation of interatomic interactions. We used the GemNet‐OC model pretrained on the OC20 dataset,^[41]^ which contains over a billion frames of data. This model comprises 38,864,438 parameters. We fine‐tuned it using our collected dataset, consisting of 18,923 frames derived from first‐principles molecular dynamics simulations of initial structures with various surface defects and cluster compositions, each running for 300 steps at 2 fs per step. The initial data used for the first fine‐tuning of the MLP model at the start of the active learning process is shown in Figure 1. During fine‐tuning, we used the Adam optimizer with a learning rate of 1e‐5 and a batch size of 12. After 40 epochs, the model achieved a mean absolute error of approximately 0.2 eV in total energy prediction on the training set. In the active learning process, newly added data constituted one‐third of each batch, ensuring a balance between old and new data when learning new data features. In each active learning cycle, we trained for 20,000 steps. The pretrained model, through its learned structural representations, accelerated convergence and enhanced the model's generalization to new structures. We found that the fine‐tuned GemNet‐OC could accurately describe our system and effectively capture complex bonding changes during Monte Carlo simulations.

To achieve rapid structure evolution, we developed a Basin‐hopping algorithm combined with MLP, where the probability of atomic displacement is directly proportional to its MLP decomposed energy. This is because atoms with higher decomposed energies, typically located in unstable environments, are more likely to undergo structural changes. The Basin‐hopping algorithm leverages these decomposed energies to efficiently identify such unstable environments and explore potential energy landscapes. This approach was integrated into the active learning process for fine‐tuning the MLP. The main steps are

(1) Initialization: Optimize the initial structure.
(2) Perturbation: Perturb the accepted structure by either perturbing all atoms with a magnitude of 0.2 Å or randomly rotating atoms around the center of two neighboring bonding atoms (based on the atom‐decomposed energies from the MLP).
(3) Local Optimization: Use the FIRE optimization algorithm ^[42]^ to optimize the test structure and obtain locally stable structures with minimal energy.
(4) Accept/Reject: Accept or reject the temporary stable structure based on the Metropolis criterion, with a kT value of 0.05 eV, and return to step (2). The choice of kT = 0.05 eV is a carefully considered compromise. While the typical CVD growth temperature of MoS_2_ (around 680°C) corresponds to a kT value of approximately 0.082 eV, we selected a slightly lower value to account for the post‐growth cooling process. This compromise value not only reflects the temperature changes during the growth process but also strikes a balance between maintaining sufficient configurational exploration and computational efficiency. Preliminary tests showed that 0.05 eV allows adequate sampling of relevant configurations while maintaining a reasonable acceptance rate of energetically favorable states.

To effectively explore the potential energy surface and improve the accuracy of the MLP, we propose an active learning framework, as illustrated in Figure S1a. The main steps are training the initial model, running the MC program to sample new structures, filtering structures based on energy, calculating energy using density functional theory (DFT), and incrementally training the model to iteratively improve its predictive performance. During incremental training, we use random sampling to select new and old data with a probability ratio of 1:4, thereby improving the efficiency of learning new data while considering the old data. We use energy as the structural identifier, and the sampling rule is to collect only one structure for each energy range, with a minimum energy interval of 0.2 eV. As shown in Figure S1b, after 10 iterations, the accuracy of the model on the test set was improved to 0.177 eV.

## 3 RESULTS AND DISCUSSION

### 3.1 S cluster interaction with MoS_2_ surfaces

**FIGURE 2:**
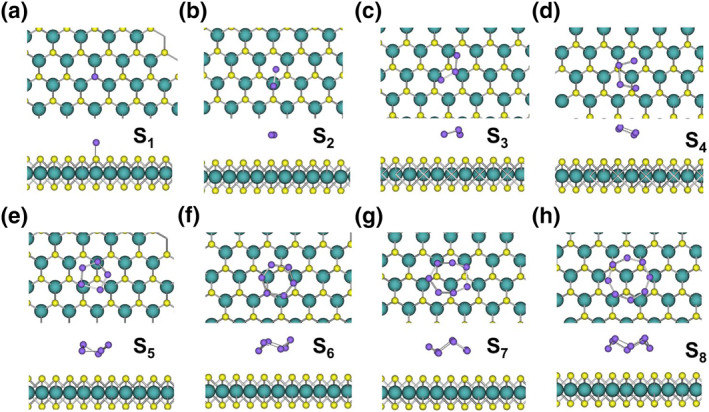
(a–h) Stable structures of S_1_ to S_8_ clusters on the perfect MoS_2_ surface (top view and side view).

We first discuss the behavior of S clusters on MoS_2_ surfaces. Figure S2a shows the MLMC energy curve of the S_1_ cluster on the perfect MoS_2_ surface, indicating minimal energy changes due to weak interactions. The initial and final structures of S_1_ and S_2_ clusters on the perfect MoS_2_ surface are depicted in Figure S2b,d. These images confirm that the S_1_ and S_2_ clusters maintain a physically adsorbed state throughout the simulation, with no significant structural changes or strong bonding to the surface. Figure 2a–h show the stable structures of S clusters on the perfect MoS_2_ surface. For S atomic clusters on the perfect MoS_2_ surface, due to the saturation of surface atoms, there are no active sites available for S atom adsorption. Thus, S clusters undergo only simple physical adsorption, and the difference between the adsorption structure on the MoS_2_ surface and in vacuum is minimal. S clusters lie flat on the perfect MoS_2_ surfaces.

In contrast to the simple physical adsorption on the perfect MoS_2_ surface, S clusters exhibit complex interactions with the Mo‐V defect. Figure S3 illustrates the MLMC energy curves and structural transitions for S_1_ and S_2_ clusters on Mo‐V in MoS_2_, capturing the initial, intermediate, and final configurations of these clusters as they interact with the defect site. Figure S3a,b show the MLMC energy curves and structural transitions for S_1_, where the S atom quickly enters the Mo‐V, reducing the energy by approximately 2.6 eV. Figure S3c,d show the MLMC energy curves and structural transitions for S_2_. During the evolution of S_2_, one S atom adsorbs next to the unsaturated S atoms near the Mo‐V, forming a metastable structure. The MLMC indicates that this configuration lowers the energy by about 1.4 eV. Subsequently, when one S atom enters the Mo‐V, the energy is further reduced by approximately 1.4 eV, totaling an energy reduction of about 2.8 eV. The evolution processes of other S clusters are similar.

**FIGURE 3:**
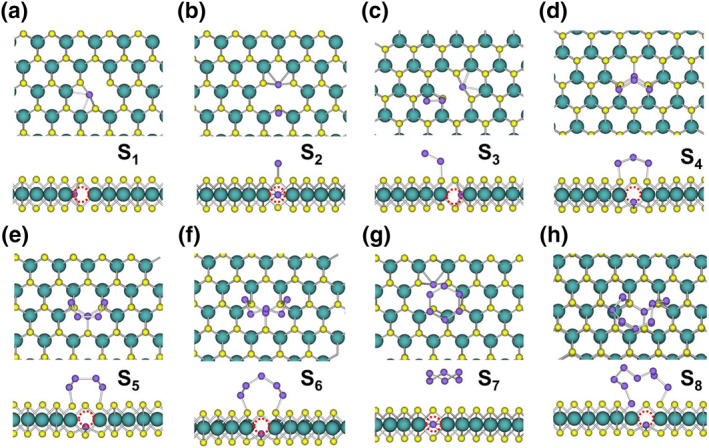
(a–h) Stable structures of S_1_ to S_8_ clusters on Mo‐V in MoS_2_ (top view and side view).

Next, we discuss the final stable structures of S_1_ to S_8_ on Mo‐V (Figure 3). Figure 3a shows the stable structure of the S_1_ cluster on the Mo‐V, where the S atom enters the vacancy and bonds with four neighboring Mo atoms. Figure 3b shows the S_2_ cluster on the Mo‐V, with one S atom entering the vacancy and the other bonding above it due to limited space. Figure 3c shows the S_3_ cluster, where one S atom enters the vacancy and the remaining two form a dimer adsorbed near the Mo‐V. Figure 3d–f,h show the S_4_, S_5_, S_6_, and S_8_ clusters, respectively, where one S atom enters the vacancy, and the rest form a bridging configuration bonding with two S atoms on one side of the Mo‐V. Figure 3g shows the S_7_ cluster, with one S atom in the vacancy and the remaining six forming a stable S_6_ ring adsorbed on the surface, due to the high stability of the S_6_ ring.

### 3.2 Mo cluster interaction with MoS_2_ surfaces

Investigating the interaction between Mo clusters and the MoS_2_ surface, we observed a previously unreported phenomenon. In contrast to prior studies where Mo atoms remained on the surface, our findings demonstrate that Mo clusters embed themselves within the MoS_2_ lattice. This embedding process is accompanied by the migration of S atoms from the Mo clusters to new surface sites, resulting in a final configuration where only S atoms are exposed on the surface. Given the significant structural rearrangements occurring during this process, a rigorous validation of the employed computational methods, particularly the MLPs, is essential. Figure S6 and Figure S7 show the energy comparison between DFT and MLP calculations for the intermediate structures obtained using the MC method, and more comparisons can be found. It can be seen that during the MC structural evolution process, the energy from MLP can well correspond to the energy from DFT. This proves the reliability of our method in exploring this process with significant structural changes.

Figure S4 presents the MLMC energy curves and structural transitions for Mo_1_ and Mo_2_ clusters on a perfect MoS_2_ surface. Figure S4a,c illustrate the energy variations throughout the simulation steps for Mo_1_ and Mo_2_, respectively, showing how the energy landscape stabilizes as the simulation progresses. The initial and final structures of these clusters, depicted in Figure S4b,d, demonstrate the dynamic changes in the cluster configurations. Initially, Mo_1_ and Mo_2_ are positioned above the surface, and through the course of the simulation, they embed into the lattice, significantly altering their configurations and lowering the system's overall energy. This embedding process is crucial for understanding the interaction dynamics and stability of Mo clusters on MoS_2_ surfaces, providing insights into potential changes in electronic properties and catalytic behaviors.

**FIGURE 4:**
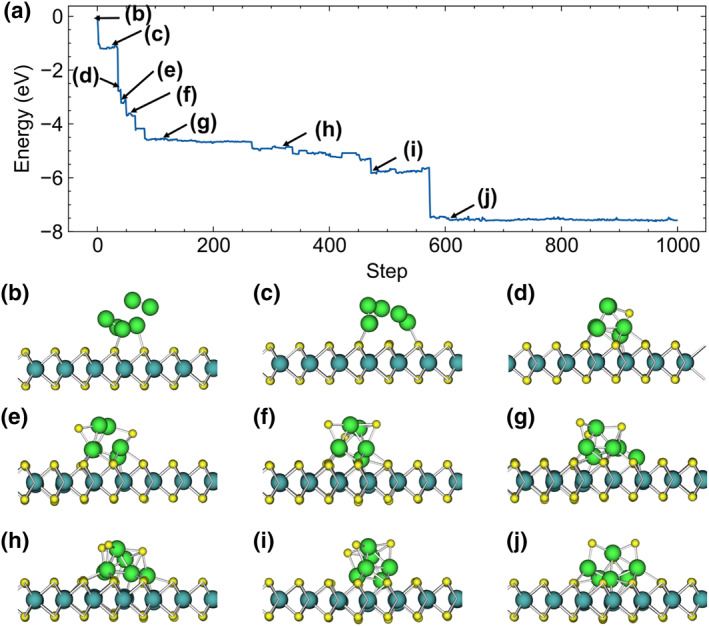
(a) Mo_6_ on perfect MoS_2_ surface MLMC energy curve. (b–j) Representative MLMC intermediate structures' side views.

For larger Mo clusters, we illustrate the MLMC energy curves and intermediate structures for Mo_6_ on a perfect MoS_2_ surface in Figure 4. As the simulation progresses, Mo_6_ clusters embed into the MoS_2_ lattice, with Mo atoms remaining within the lattice, while S atoms migrate to new surface sites. The energy curve in Figure 4a shows significant energy drops at specific steps, corresponding to the structural transitions depicted in Figure 4b–j. Each drop in the energy curve indicates a stabilization phase where the Mo_6_ cluster undergoes a configurational change that results in a more energetically favorable state. Figure 4b shows the initial state of the Mo_6_ cluster lying on the MoS_2_ surface, with the cluster maintaining a configuration similar to its vacuum state. As the MLMC progresses to the state shown in Figure 4c, the Mo_6_ cluster becomes flatter, with more Mo atoms coming closer to the MoS_2_ surface. In Figure 4d, the first S atom leaves the MoS_2_ surface and moves to the top of the Mo_6_ cluster. Subsequently, in Figure 4e, two S atoms are pulled out, and in Figure 4f, three S atoms are pulled out. In the states shown in Figure 4g–j, although no more S atoms are pulled out, the Mo_6_ cluster embedded in the MoS_2_ surface still undergoes drastic structural changes, leading to further energy reduction. After reaching the structure shown in Figure 4j, we performed over 400 more MLMC steps, and the structure remained largely unchanged. The behavior of other larger Mo clusters is similar.

**FIGURE 5:**
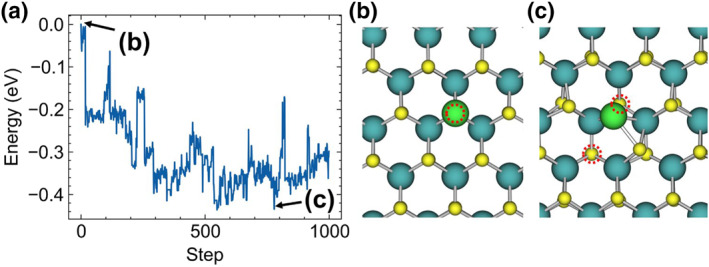
(a) Mo_1_ on S_2_‐V in MoS_2_ surface MLMC energy curve. (b) Init and (c) final structure of Mo_1_ on S_2_‐V in MoS_2_ surface.

Next, we explored the behavior of Mo clusters near the S_2_‐V defect on the MoS_2_ surface. Figure 5 illustrates the evolution process of a single Mo atom (Mo_1_) on the MoS_2_ surface containing an S_2_‐V. The MLMC energy curve in Figure 5a captures the energy changes during the structural evolution. In the initial state, shown in Figure 5b, the Mo atom adsorbs at the original position of the missing S atom on the upper surface of the S_2_‐V. As the MLMC simulation progresses, the system explores various configurations to minimize its energy. The final state identified using the MLMC process, shown in Figure 5c, reveals an intriguing structural rearrangement. The remaining S atoms on the upper surface migrate to occupy one of the positions in the S_2_‐V. At the same time, the Mo atom replaces an S atom, effectively creating a new defect configuration. The resulting structure features a single S‐V and a unique defect where the Mo atom substitutes an S atom on the upper surface. The entire process only results in an energy decrease of approximately 0.5 eV. Figure S5 illustrates the evolution process of Mo_2_ on the MoS_2_ surface containing an S_2_‐V. The MLMC energy curve in Figure S5a captures the energy changes during the structural evolution. In the initial state, shown in Figure S5b, the Mo_2_ adsorbs at the original position of the missing S atom on the upper surface of the S_2_‐V. Then, like the case of Mo1, there will be a S atom entering the other side to fill the S‐V, resulting in the formation of a single S‐V and a defect where Mo_2_ substitutes S on the upper surface. In the final state identified using the MLMC process, shown in Figure S5c, one Mo atom in Mo2 will fill another remaining S‐V. Larger Mo clusters exhibit similar behavior, and next, we will discuss the stable structures following these evolutionary processes.

**FIGURE 6:**
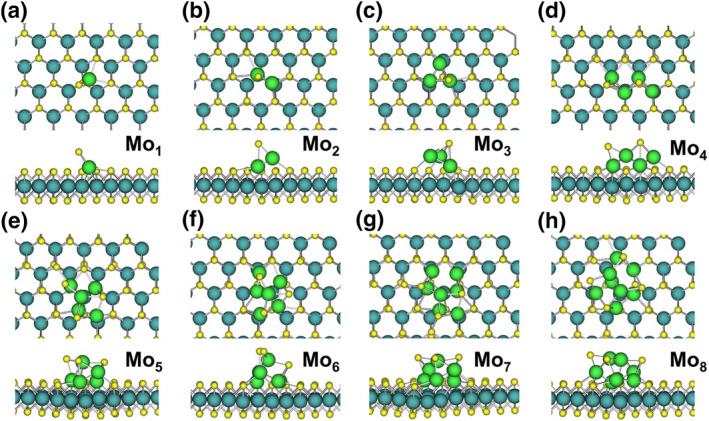
(a–h) Final stable structures of Mo_1_–Mo_8_ clusters embedded on the perfect MoS_2_ surface.

The stable structures of Mo_1_ to Mo_8_ clusters on the perfect MoS_2_ surface, as depicted in Figure 6, reveal a clear trend: increasing cluster size leads to stronger interactions with the surface and more pronounced structural modifications. For a single Mo atom (Mo_1_), embedding occurs with the displacement of one surface S atom (Figure 6a). The displaced S atom forms a bond with the embedded Mo atom (2.13 Å), significantly shorter than the typical Mo‐S bond length in MoS_2_ (2.40 Å). The embedded Mo atom forms two equal‐length bonds with the S atoms in the layer, with a bond length of 2.47 Å. As the cluster size increases to Mo_2_ and Mo_3_, only one S atom displacement is observed (Figure 6b–c). However, for Mo_4_, two surface S atoms are displaced (Figure 6d). This trend continues with Mo_5_ displacing three S atoms and lowering the energy by 5.9 eV (Figure 6e). Mo_6_ and Mo_7_ clusters also displace three S atoms each, respectively (Figure 6f–g). Finally, the Mo_8_ cluster displaces four S atoms (Figure 6h). In all cases, the Mo clusters embed within the subsurface of MoS_2_, leaving only S atoms exposed on the surface. This embedding behavior and the associated structural changes contribute significantly to the overall energy reduction observed in these systems.

Based on our previous research and analysis, we propose that the “surface sulfur passivation” mechanism can explain the process of molybdenum cluster embedding into the molybdenum disulfide (MoS_2_) surface. This mechanism is driven by the system's inherent tendency towards energy minimization and chemical stability, manifesting as sulfur atoms consistently tending to occupy surface positions to passivate underlying molybdenum atoms. When a molybdenum cluster comes into contact with the MoS_2_ surface, the surface lacks sufficient sulfur atoms to passivate all molybdenum atoms. To achieve complete passivation, some surface sulfur atoms migrate from their original positions to the top of the molybdenum cluster, while molybdenum atoms move downward, gradually embedding into the MoS_2_ lattice. During this process, the system continuously adjusts the positions of molybdenum and sulfur atoms, always maintaining sulfur atoms on the outermost layer until a stable, energy‐minimized configuration is formed. This mechanism not only explains the embedding behavior of molybdenum clusters but also reveals the self‐organizing capability of MoS_2_ materials at the atomic scale, maintaining surface stability through the rearrangement of molybdenum and sulfur atoms.

**FIGURE 7:**
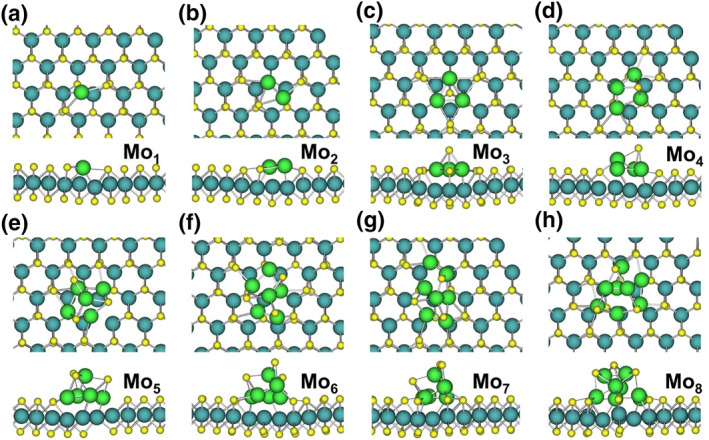
(a–h) Final stable structures of Mo_1_–Mo_8_ clusters embedded on the MoS_2_ surface with Mo‐V.

The interaction of Mo clusters with a defective MoS_2_ surface containing S_2_‐V, as illustrated in Figure 7, exhibits distinct characteristics compared to the perfect surface. Smaller Mo_1_ and Mo_2_ clusters preferentially adsorb near the S_2_‐V without displacing surface S atoms (Figure 7a–b), indicating that the vacancy acts as favorable adsorption sites. As cluster size increases to Mo_3_ and Mo_4_, embedding occurs with the displacement of one surface S atom (Figure 7c–d), suggesting that the S_2_‐V facilitates integration into the MoS_2_ lattice. For larger clusters (Mo_5_ to Mo_8_), embedding involves the displacement of three surface S atoms (Figure 7e–h). While similar to the behavior observed on the perfect surface, the presence of the S_2_‐V appears to enhance structural rearrangement and facilitate the embedding process. The S_2_‐V not only acts as preferential adsorption sites for smaller Mo clusters but also promotes the integration of larger clusters into the MoS_2_ lattice. This interaction potentially leads to the stabilization of novel defect structures with altered properties.

Mo clusters have a profound impact on MoS_2_ surfaces, and modifying the MoS_2_ surface by embedding Mo clusters can influence its electronic properties, which may be beneficial for the development of advanced electronic devices such as transistors and sensors. Moreover, unraveling these interactions can provide valuable insights for reactions such as hydrogen evolution and CO_2_ reduction, as well as the design of novel and efficient single‐atom catalysts.

### 3.3 Relative stability of S and Mo clusters on MoS_2_ surfaces

To assess the relative stability of different cluster configurations, we calculated the average formula energy using the equation:

$$\frac{E}{n}=\frac{\left(E_{0}-E_{\text{substrate}}\right)}{n},$$

where *E*_substrate_ is the energy of the substrate and *E*_0_ is the energy of the stable structure of substrate with the defect.

**FIGURE 8:**
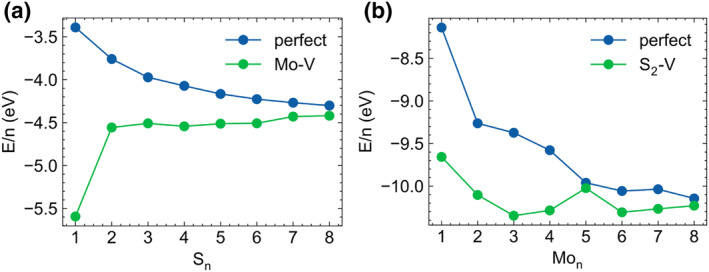
(a) Average formula energy of S cluster in MoS_2_ surface (perfect surface and Mo‐V defect). (b) Average formula energy of Mo cluster in MoS_2_ surface (perfect surface and Mo‐V defect).

Figure 8a shows the average formula energy of different S clusters on the perfect MoS_2_ surface and the Mo‐V defect surface. For the perfect surface, the S_8_ cluster has the lowest formation energy, as the S_8_ ring is the most stable form of S in the S‐rich environment. However, on the Mo‐V defect surface, the S_1_ cluster structure resulting from the evolution of the vacancy has the lowest free energy, indicating it is the most stable configuration. Figure 8b shows the average formula energy of Mo clusters on the perfect MoS_2_ surface and the S_2_‐V defect surface. On the perfect surface, the average formation energy increases with the size of the Mo cluster. On the surface with the S_2_‐V defect, the Mo_3_ cluster exhibits the lowest formation energy, as shown in Figure 7c. In this structure, Mo atoms replace three S atoms on the upper surface, and the newly elevated S atom is positioned directly above these three Mo atoms, forming a highly symmetrical structure.

## 4 CONCLUSION

This work has provided a comprehensive analysis of the interactions between Mo and S clusters with MoS_2_ surfaces, employing a combination of MLPs and MC simulations. Our findings reveal distinct behaviors of these clusters on both perfect and defective MoS_2_ surfaces, which are critical for understanding the fundamental aspects of material behavior during the CVD growth process.

We observed that S clusters tend to exhibit only mild physical adsorption on perfect MoS_2_ surfaces, indicating minimal interaction. However, Mo clusters, due to their high surface energy, embed themselves into the MoS_2_ lattice, creating new defect structures that are challenging to reverse. This behavior underscores the importance of controlling Mo cluster formation during synthesis to maintain the integrity of the MoS_2_ surfaces. On defective MoS_2_ surfaces, the behavior of these clusters becomes more complex. S clusters can fill vacancies, bonding with unsaturated S atoms, while Mo clusters continue to embed themselves, similar to their behavior on perfect surfaces. This suggests that Mo clusters have a robust interaction mechanism with the MoS_2_ surface that is largely unaffected by the presence of defects. The implications of our study are significant for the synthesis and application of 2D materials. By understanding the interaction dynamics of clusters with MoS_2_ surfaces, we can better tailor the properties of these materials for advanced applications in electronics, optoelectronics, and catalysis.

## CONFLICT OF INTEREST STATEMENT

The authors declare no conflicts of interest.

## ETHICS STATEMENT

The research did not include any human or animal experiments.

## Supporting information

Supporting Information S1

## Data Availability

Data available on request from the authors.
