# Supplementary material for: Unveiling the unexpected sinking and embedding dynamics of surface supported Mo/S clusters on 2D MoS2 with active machine learning
Source: Smart Mol. 2024 Aug 8;3(1):e20240018. doi: 10.1002/smo.20240018 (PMC12117921; doi:10.1002/smo.20240018)
Supplement: Supplementary file 1 — Supporting Information S1 [file SMO2-3-e20240018-s001.docx]

**Unveiling the Unexpected Sinking and Embedding Dynamics of Surface Supported Mo/S Clusters on 2D MoS_2_ with Active Machine Learning**

Luneng Zhao^1^, Yanhan Ren^1^, Xiaoran Shi^1^, Hongsheng Liu^1^, Zhigen Yu^2^, Junfeng Gao^1^*, Jijun Zhao^1^

1.State Key Laboratory of Structural Analysis for Industrial Equipment&School of Physics, Dalian University of Technology, Dalian, 116024 China

2.Institute of High Performance Computing (IHPC), Agency for Science, Technology and Research(A*STAR), 1 Fusionopolis Way, #16-16 Connexis, Singapore 138632, Republic of Singapore

# Supporting Information


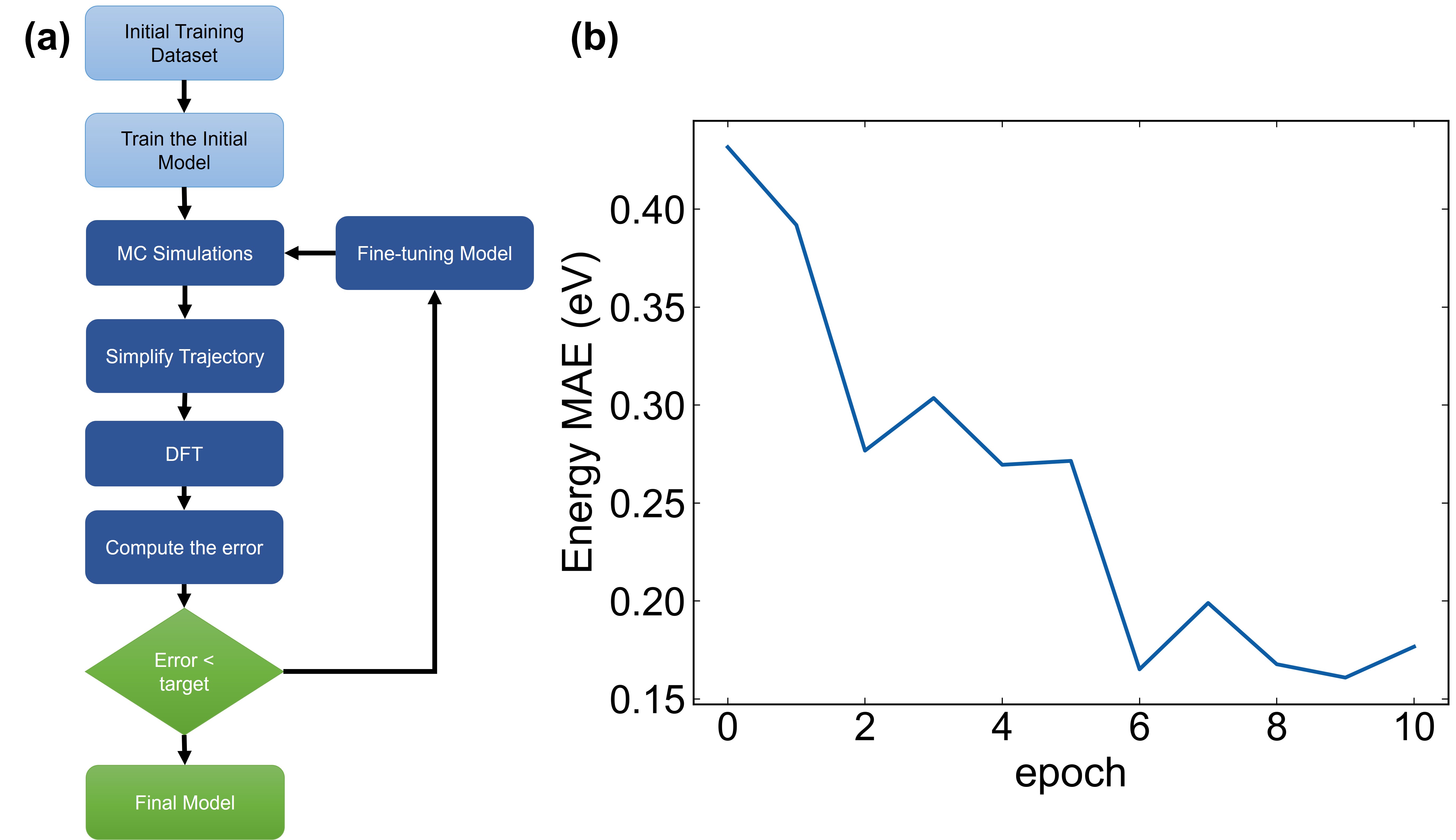


**FIG. S1.: Active learning framework and model performance.** (a) Flowchart of the active learning framework for iteratively improving the machine learning potential model. (b) MAE in energy prediction on the test set as a function of the active learning iteration.


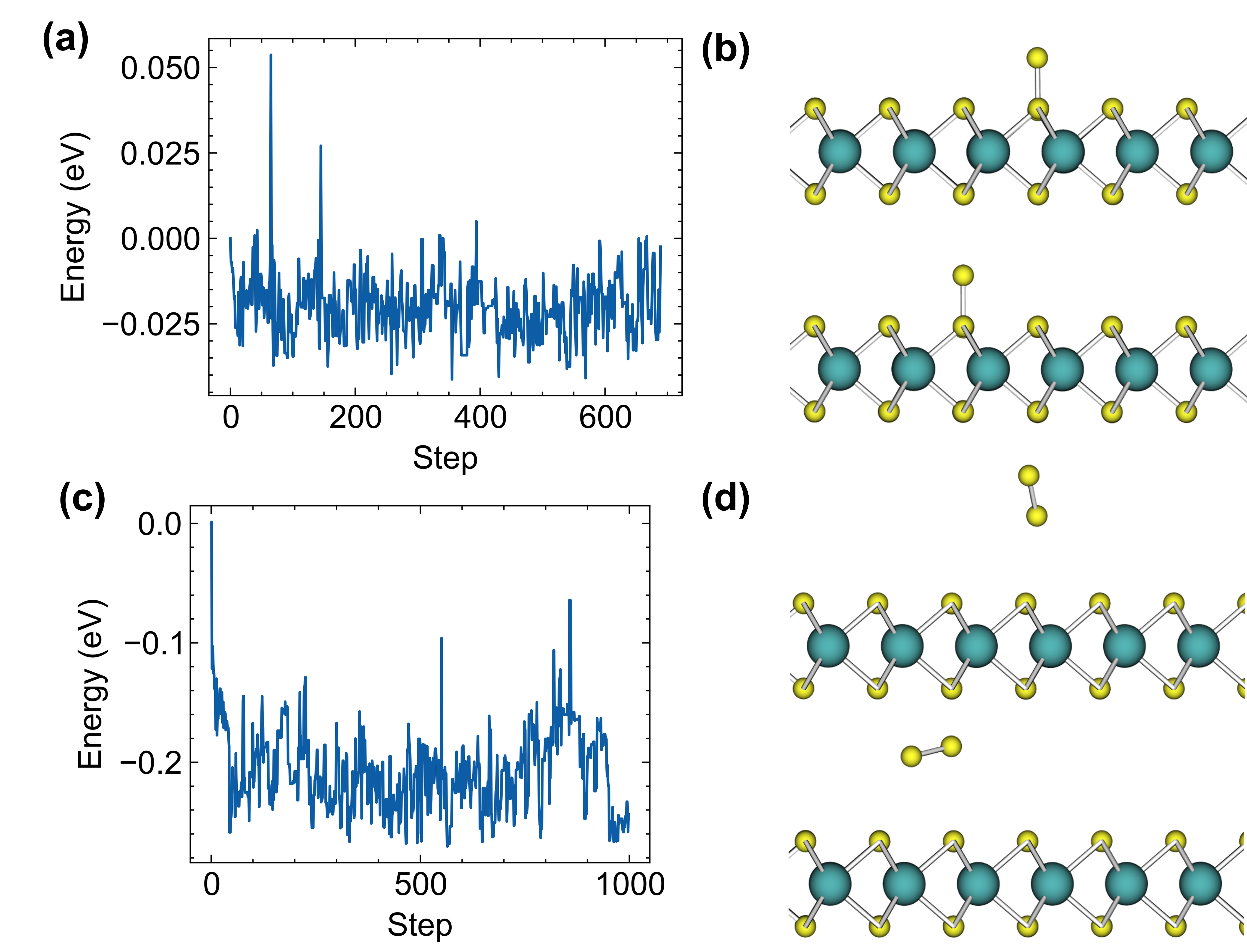


FIG. S2.: (a) S_1_ on perfect MoS_2_ surface MLMC energy curve. (b) S_1_ in perfect MoS_2_ surface init structure (top) and final structure (bottom). (c) S_2_ on perfect MoS_2_ surface MLMC energy curve. (d) S_2_ on perfect MoS_2_ surface init structure (top) and final structure (bottom).


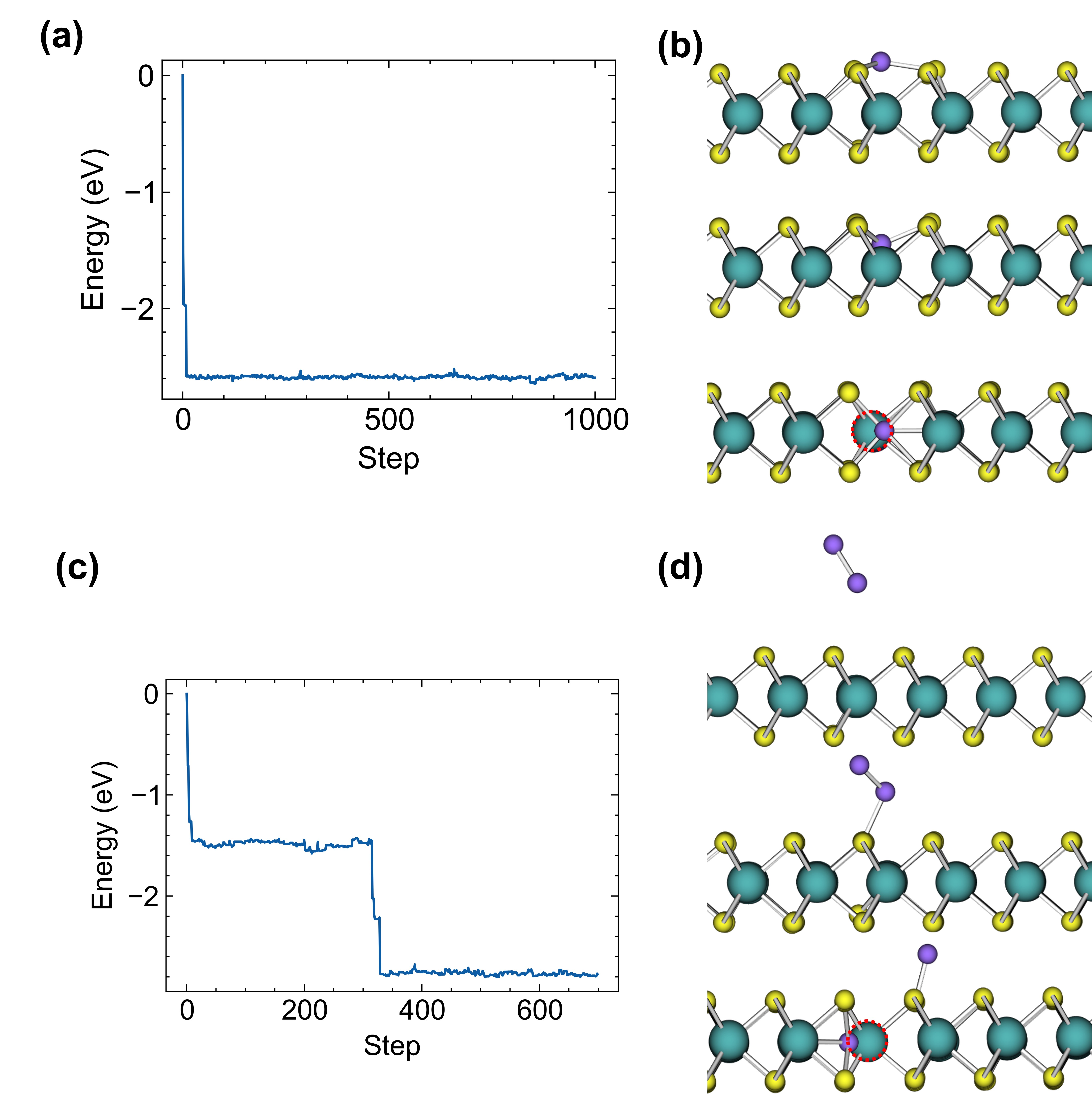


FIG. S3.: (a) S_1_ on Mo-V in MoS_2_ surface MLMC energy curve. (b) S_1_ in Mo-V init structure (top) , intermediate structure (middle) and final structure (bottom). (c) S_2_ on Mo-V in MoS_2_ surface MLMC energy curve. (d) S_2_ in Mo-V init structure (top) , intermediate structure (middle) and final structure (bottom).


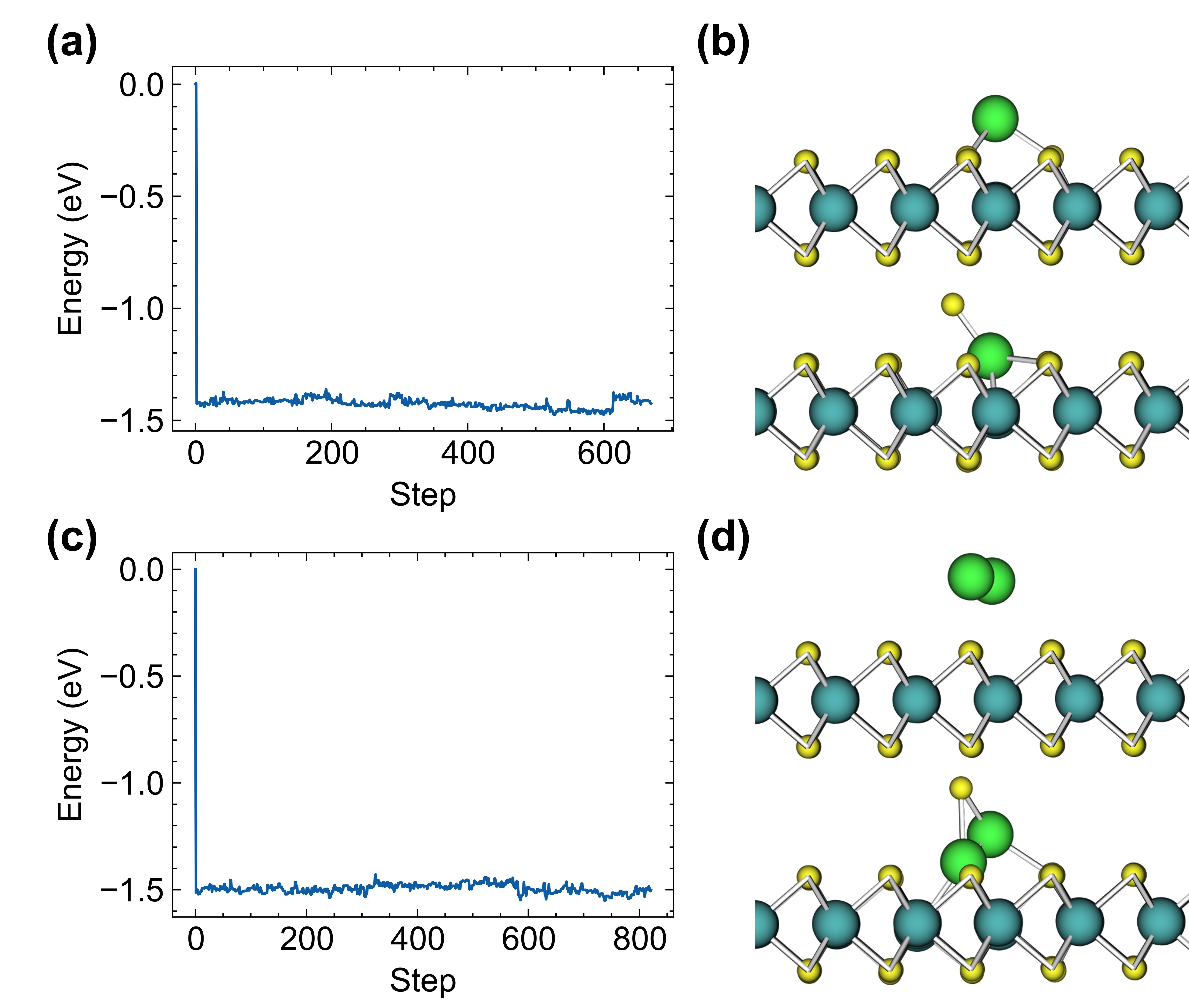


FIG. S4.: (a) Mo_1_ on perfect MoS_2_ surface MLMC energy curve. (b) Mo_1_ in perfect MoS_2_ surface init structure (top) and final structure (bottom). (c) Mo_2_ on perfect MoS_2_ surface MLMC energy curve. (d) Mo_2_ in perfect MoS_2_ surface init structure (top) and final structure (bottom).


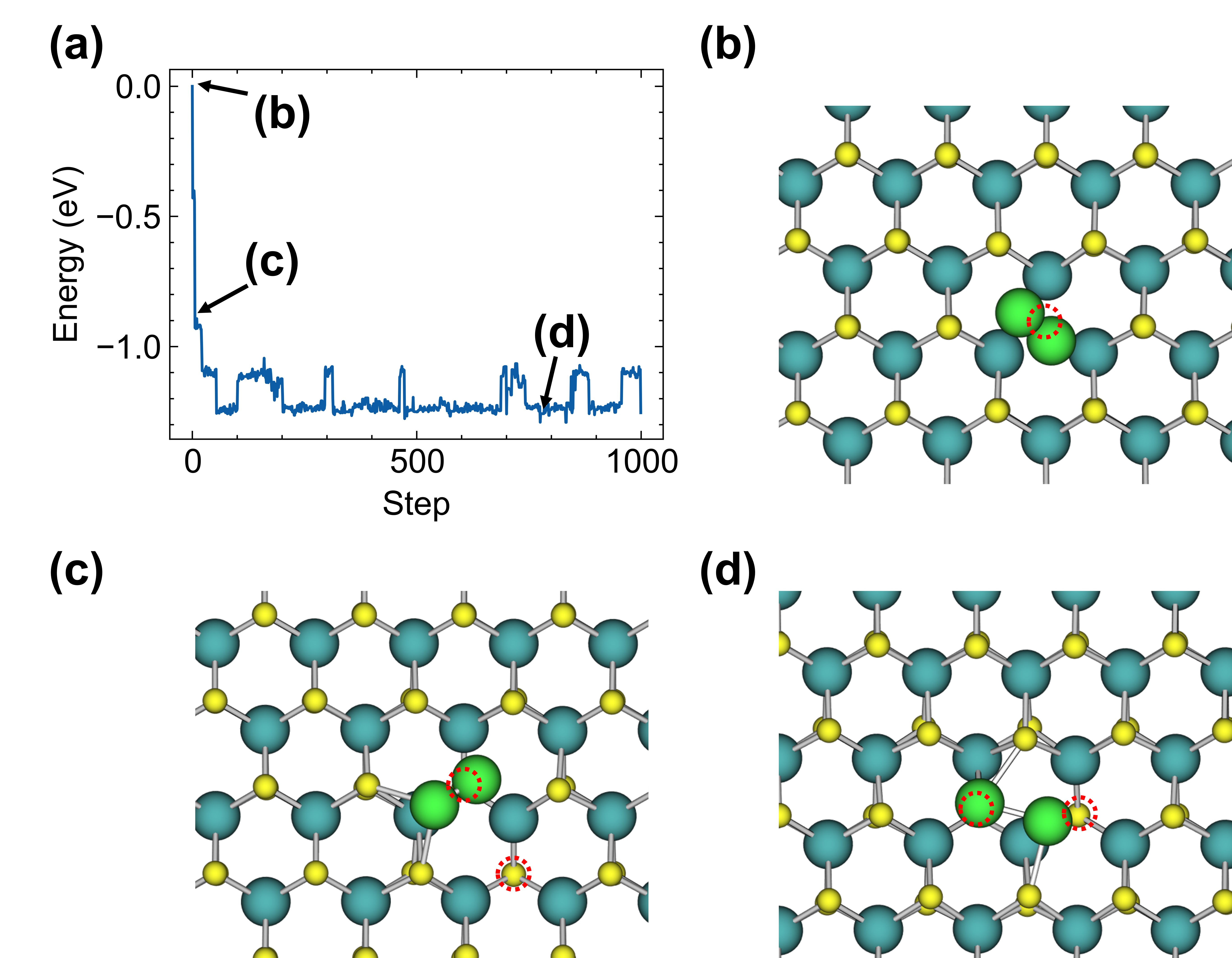


FIG. S5.: (a) Mo_2_ on S_2_-V in MoS_2_ surface MLMC energy curve. (b) Init , transition(c) and (d) final structure of Mo_2_ on S_2_-V in MoS_2_ surface.


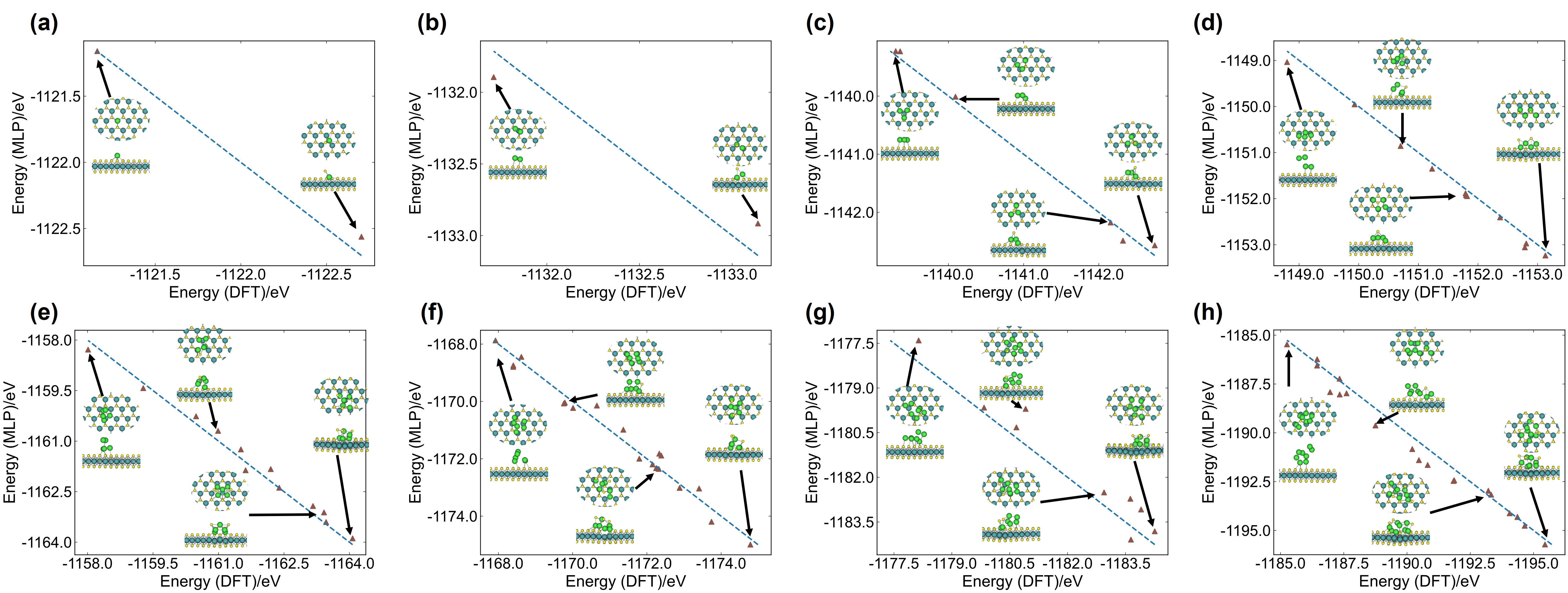


FIG. S6.: (a-h) Structural evolution of Mo_1_-Mo_8_ on perfect MoS_2_ surface, and the relationship between energy calculated by DFT and energy calculated by MLP.(green and blue atom is Mo)


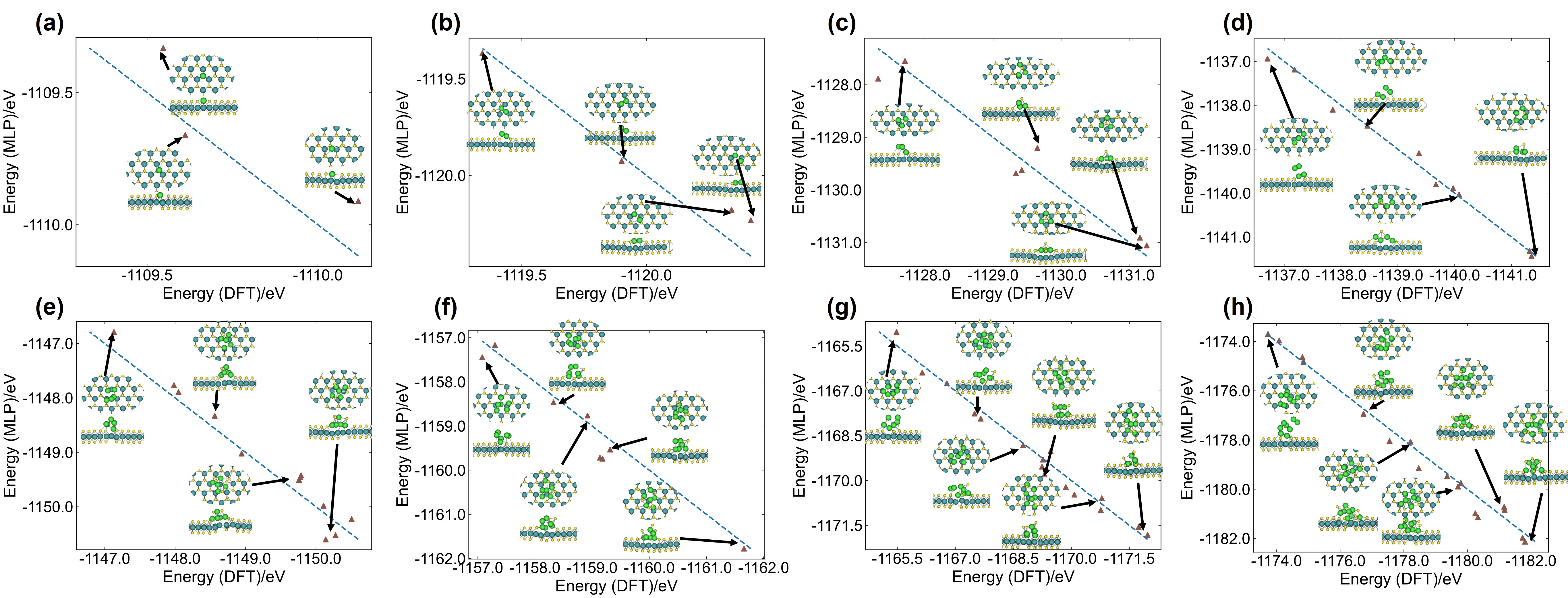


FIG. S7.: (a-h) Structural evolution of Mo_1_-Mo_8_ on S_2_-V in MoS_2_, and the relationship between energy calculated by DFT and energy calculated by MLP. (green and blue atom is Mo)
